# MicroRNAs as novel biomarkers for prenatal fetal congenital heart disease – systematic literature review

**DOI:** 10.3389/fgene.2025.1628632

**Published:** 2025-07-30

**Authors:** Adrianna Kondracka, Paulina Gil-Kulik, Magda Rybak-Krzyszkowska, Jakub Staniczek, Anna Oniszczuk, Bartosz Kondracki

**Affiliations:** ^1^ Department of Obstetrics and Pathology of Pregnancy, Medical University of Lublin, Lublin, Poland; ^2^ Department of Clinical Genetics, Medical University of Lublin, Lublin, Poland; ^3^ Department of Obstetrics, Perinatology University Hospital, Kraków, Poland; ^4^ Chair and Department of Gynecology, Obstetrics and Gynecological Oncology, Medical University of Silesia, Katowice, Poland; ^5^ Department of Inorganic Chemistry, Medical University of Lublin, Lublin, Poland; ^6^ Department of Cardiology, Medical University of Lublin, Lublin, Poland

**Keywords:** congenital heart defects (CHD), microRNAs (miRNAs), biomarkers, prenatal diagnosis, fetal echocardiography, maternal serum, amniotic fluid, umbilical cord blood

## Abstract

**Introduction:**

CHD accounts for about one-third of all congenital malformations and is the leading cause of infant mortality. Currently, the primary method for diagnosing CHD during pregnancy is fetal echocardiography. Several studies have observed significant differences in the expression levels of specific miRNAs between CHD fetuses and normal fetuses. This systematic review explores the potential of miRNAs as non-invasive biomarkers for the prenatal detection of CHD in fetuses.

**Material and methods:**

The systematic review followed PRISMA guidelines, conducting a detailed search across PubMed, Scopus, and Web of Science using predefined terms related to microRNAs and congenital heart defects. Inclusion was limited to original, full-text articles in English, while non-English studies, reviews, and inaccessible full texts were excluded. Data extraction and quality assessment using the Newcastle Ottawa Scale ensured comprehensive evaluation and minimized bias.

**Results:**

Studies explored the potential of miRNAs as biomarkers for detecting congenital heart defects (CHD) in fetuses, employing diverse sample types such as maternal serum, umbilical cord blood, and amniotic fluid. Diagnostic methods primarily included fetal echocardiography, complemented by postnatal confirmation through surgery or autopsy. Gestational ages at sample collection ranged predominantly from the second trimester (16–27 weeks) to narrower windows, reflecting methodological variability across studies. The included studies utilized advanced technologies, such as next-generation sequencing (e.g., Illumina HiSeq, NovaSeq) and microarrays, for discovery-phase experiments, while validation predominantly employed qRT-PCR techniques. Identified miRNAs showed heterogeneity in expression patterns and diagnostic potential, with several studies reporting high sensitivity, specificity, and AUC values for specific miRNAs like miR-146a-5p and miR-142-5p. While some miRNAs demonstrated exceptional diagnostic accuracy, others were only described in terms of differential expression, highlighting the variability and complexity of miRNA biomarker discovery for CHD.

**Conslusion:**

The findings of this systematic literature review provide evidence that some miRNAs could serve as non-invasive biomarkers for the early detection of CHD in fetuses. However, each of the reviewed studies identified different miRNAs as potential biomarkers. This variability may stem from differences in experimental methodologies, including approaches to miRNA isolation, quantification techniques, and the types of biological materials analyzed. Such methodological heterogeneity, combined with small sample sizes and the diverse spectrum of CHDs, underscores the need for caution in interpreting these findings. At this stage, it is not feasible to translate these results into clinical practice or establish standardized miRNA-based prenatal screening protocols.

## 1 Introduction

CHD accounts for about one-third of all congenital malformations and is the leading cause of infant mortality. CHD is one of the most common birth defects, significantly impacting the structure and function of the heart and its vessels and posing a serious complication during pregnancy. Some forms of CHD include ventricular and atrial septal defects (VSD and ASD respectively), tetralogy of fallot (ToF), coarctation of aorta (CoA). It is estimated that the global prevalence of CHD was 1.8 cases per 100 live births, in 2017. The annual global mortality was estimated to be 261,247 in 2017. Of all the mortality cases 180,624 (69.14%) occurred in infants younger than 1 year (GBD, 2017 Congenital Heart Disease Collaborators, 2020). In cases of fetal deaths, the incidence of CHD is associated with the gestational age at which the fetal loss occurs. Early diagnosis of CHD is crucial for better prognosis and timely surgical intervention, leading to improved outcomes for both the mother and the fetus (Eckersley et al., 2016).

Currently, the primary method for diagnosing CHD during pregnancy is fetal echocardiography. Fetal echocardiography is typically performed between 22 and 28 weeks of pregnancy. During this period, detailed fetal heart structure imaging helps identify abnormalities. However, echocardiography has limitations. The accuracy of diagnosis depends on the skill of the operator performing the echocardiogram. The quality of the ultrasound equipment used affects the diagnostic accuracy. A lack of standardized processes can impact consistency in CHD detection (Chew et al., 2007). Recent research has focused on identifying early biomarkers for CHD in maternal blood. While biomarkers like elevated nuchal translucency (NT), Free β hCG, and lowered pregnancy-associated plasma protein A are not specific enough for accurate fetal CHD screening, microRNAs have emerged as promising disease biomarkers (Martínez-Moratalla et al., 2012; Hunter and Simpson, 2014).

MicroRNAs (miRNAs), discovered in 1993 in *Caenorhabditis elegans*, are small non-coding RNA fragments (17–25 nucleotides long) highly conserved across various species. These miRNAs play a crucial role in gene regulation by inhibiting protein translation, affecting mRNA expression, and inducing mRNA degradation. They are found in serum, plasma, amniotic fluid, and body tissues. MiRNAs influence various biological processes such as cell proliferation, differentiation, apoptosis, immune response, stem cell growth, aging, and haematopoiesis. Due to their broad regulatory roles and stability in biological fluids, miRNAs hold significant potential as diagnostic markers for various diseases (Pang et al., 2019).

These miRNAs can be used also as prenatal biomarkers for CHD (Song et al., 2018). Their ability to cross the placental barrier and their stability in maternal circulation make them promising candidates for accurate CHD screening during pregnancy (Pasławska et al., 2024). Several studies have observed significant differences in the expression levels of specific miRNAs between CHD fetuses and normal fetuses (Zhu et al., 2013; Kehler et al., 2015; Gu et al., 2019; Jin et al., 2021; Kong et al., 2021; Yang et al., 2022; Xi et al., 2024). Measuring the levels of circulating miRNAs offers several advantages. Circulating miRNAs can be detected non-invasively from blood samples, making it a safe and convenient method for assessing various conditions. Unlike invasive procedures, such as tissue biopsies, the measurement of circulating miRNAs can be performed on samples like maternal blood, which are generally safe to collect.

This systematic review explores the potential of miRNAs as non-invasive biomarkers for the prenatal detection of CHD in fetuses. By synthesizing data from previous studies, it aims to provide a comprehensive evaluation of miRNAs’ diagnostic utility. Through the analysis of various miRNAs, this review examines their role in improving the accuracy and effectiveness of CHD screening during pregnancy.

## 2 Methods

### 2.1 Search strategy and databases search

This systematic review followed the Preferred Reporting Items for Systematic Reviews and Meta-Analyses (PRISMA) guidelines. A comprehensive literature search was conducted across three major electronic databases: PubMed, Scopus, and Web of Science. The search strategy was meticulously developed based on preliminary scoping reviews and expert knowledge within the field, ensuring the inclusion of relevant terms and keywords. Boolean operators (AND, OR) were employed to combine the search terms: mirnas, microRNA, miRNA, Heart Defects, Congenital, congenital heart disease, ventricular septal defects, atrial septal defects, Patent ductus arteriosus, Tetralogy of Fallot, prenatal diagnosis, Prenatal Diagnosis, Noninvasive Prenatal Testing. Detailed search strings used in each database are shown in Table 1. Although this review followed the PRISMA 2020 methodology, a formal meta-analysis was not performed due to substantial heterogeneity in study design, miRNA detection methods, sample types, outcome definitions, and reporting formats. Instead, a structured narrative synthesis was conducted in accordance with current best practices for systematic reviews where meta-analysis is not feasible.

**TABLE 1:** Search strings are used to search databases.

Database	Search strings
PubMed n = 12	(mirnas [MeSH Terms] OR (microRNA [Title/Abstract]) OR (miRNA [Title/Abstract])) AND ((“Heart Defects, Congenital” [Mesh] OR (congenital heart disease [Title/Abstract]) OR (ventricular septal defects [Title/Abstract]) OR “atrial septal defects” [Title/Abstract]) OR (Patent ductus arteriosus [Title/Abstract]) OR (Tetralogy of Fallot [Title/Abstract])) AND ((prenatal diagnosis [Title/Abstract) OR “Prenatal Diagnosis” [Mesh] OR “Noninvasive Prenatal Testing” [Mesh])
Scopus n = 17	(TITLE-ABS-KEY (mirnas) OR TITLE-ABS-KEY (microRNA) OR TITLE-ABS-KEY (miRNA)) AND (TITLE-ABS-KEY (“Heart Defects, Congenital”) OR TITLE-ABS-KEY (“congenital heart disease”) OR TITLE-ABS-KEY (“ventricular septal defects”) OR TITLE-ABS-KEY (“atrial septal defects”) OR TITLE-ABS-KEY (“Patent ductus arteriosus”) OR TITLE-ABS-KEY (“Tetralogy of Fallot”)) AND (TITLE-ABS-KEY (“prenatal diagnosis”) OR TITLE-ABS-KEY (“Prenatal Diagnosis”) OR TITLE-ABS-KEY (“Noninvasive Prenatal Testing”))
Web Of Science n = 7	(TS=(mirnas) OR TS=(microRNA) OR TS=(miRNA)) AND (TS=(“Heart Defects, Congenital”) OR TS=(“congenital heart disease”) OR TS=(“ventricular septal defects”) OR TS=(“atrial septal defects”) OR TS=(“Patent ductus arteriosus”) OR TS=(“Tetralogy of Fallot”)) AND (TS=(“prenatal diagnosis”) OR TS=(“Prenatal Diagnosis”) OR TS=(“Noninvasive Prenatal Testing”))

### 2.2 Inclusion and exclusion criteria

Only original research articles published in English and available in full-text format were included in the review. Publications that did not meet these criteria were excluded. Specifically, review articles, case reports, case series, opinion pieces, short communications, and conference abstracts were not considered for inclusion. Additionally, studies published in languages other than English or those for which the full text was inaccessible were excluded.

### 2.3 Selection process

Study selection was performed in two phases. In the first phase, two authors independently screened the titles and abstracts of identified articles. Articles that did not apply to the research question were excluded. Abstracts that appeared eligible for full-text screening were retrieved. In the second phase, full-text articles were thoroughly assessed to determine whether they met the predefined inclusion and exclusion criteria. Two authors independently reviewed the full texts, and any discrepancies regarding eligibility were resolved through discussion or, when necessary, by consultation with a third author. This two-step process was conducted using Zotero (ver. 6.0.36) open-source software to facilitate the organization and management of references.

### 2.4 Data collection and synthesis approach

Data from studies meeting the inclusion criteria were extracted into a structured Excel spreadsheet by two independent reviewers. The following variables were collected: author, year, study design, study population, gestational age at sampling, CHD subtype, diagnostic methods, and miRNAs investigated. Performance indicators such as AUC, sensitivity, and specificity were also recorded when available.

In addition, we extracted methodological details relevant to data heterogeneity, including type of sample (e.g., maternal serum, amniotic fluid), type of experiment (discovery vs. targeted), discovery platform (e.g., NGS, microarray), validation method (e.g., qRT-PCR), dye system used, and normalization strategy (e.g., U6, cel-miR-39).

Due to heterogeneity in CHD classification, timing of sample collection, and technological platforms, pooling of data for meta-analysis was not appropriate. Therefore, results were summarized using a narrative synthesis approach, structured around clinical and experimental characteristics. Two detailed summary tables were created to support transparency and reproducibility: one presenting the clinical characteristics and study designs, and the second presenting technical and methodological variables.

### 2.5 Study risk of bias assessment

This study used the Newcastle Ottawa Scale (NOS) to assess the potential for bias in observational studies. The overall risk of bias was categorized as low, moderate or high risk of bias. The quality of the individual studies was analysed and evidence certainty was measured indicating a lower bias (Lo et al., 2014).

## 3 Results

### 3.1 Literature search

**FIGURE 1:**
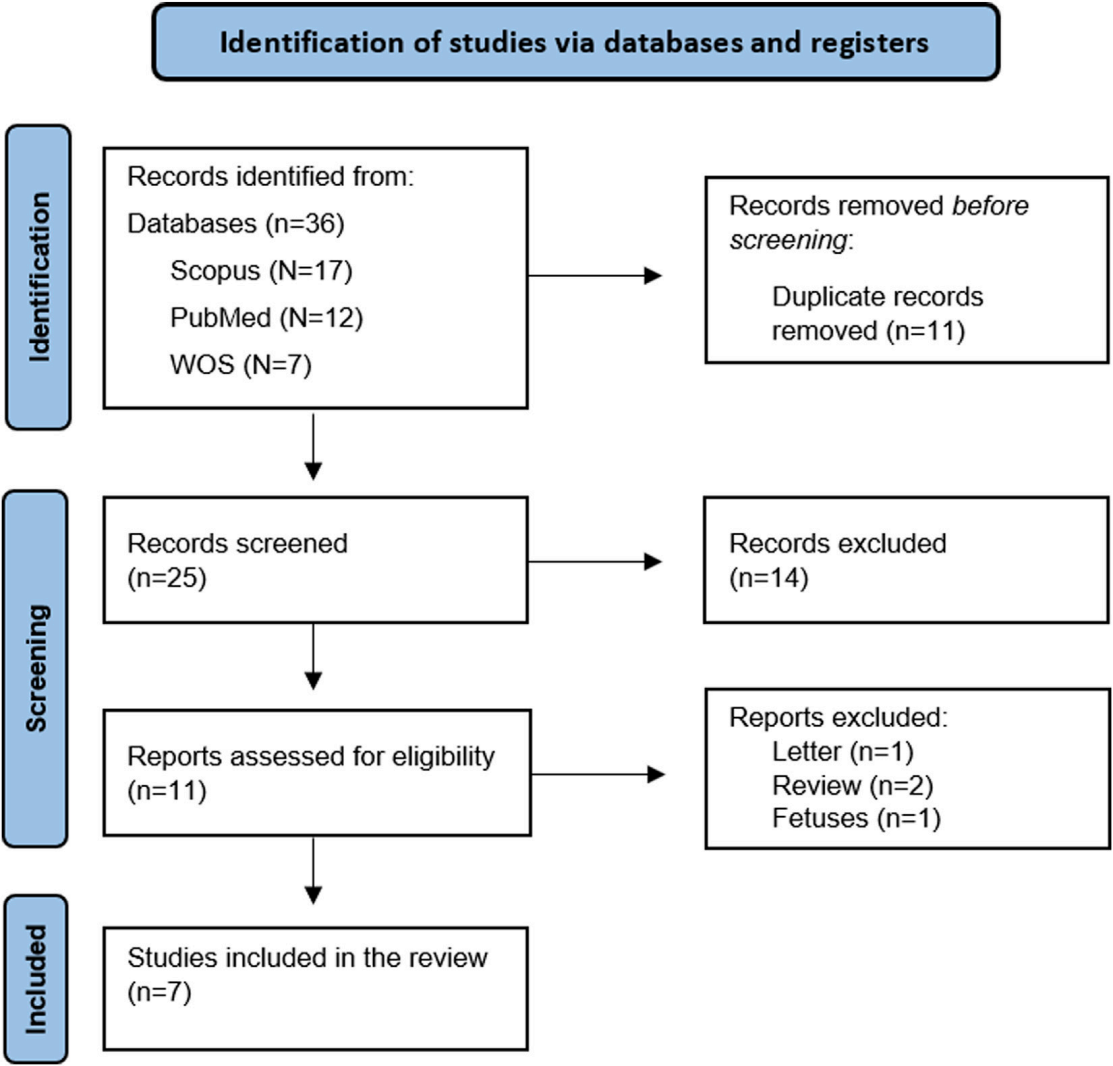
Prisma flowchart.

A total of 36 articles were identified through database searches. After removing duplicates, 25 articles remained for title and abstract screening. Of these, 11 articles were deemed eligible for full-text review. After a comprehensive assessment of the full texts against the predefined inclusion and exclusion criteria, seven articles met the eligibility requirements and were included in the systematic review. (Figure 1).

### 3.2 Overview of study characteristics of the included studies

The potential of miRNAs as biomarkers for detecting CHD in fetuses has been identified in seven studies included in this review. The studies included in this systematic review featured diverse designs and methodologies. The sample sizes ranged from 14 to 182 participants, with a balanced distribution of study and control groups across most studies. Diagnostic methods for CHD predominantly relied on fetal echocardiography, supplemented by postnatal surgery or autopsy in some studies. Among the CHD types, VSD was the most frequently studied, with some investigations also addressing TOF, persistent truncus arteriosus (PTA), single ventricle (SV), transposition of the great arteries (TGA), and other mixed or rare defects. Gestational age at sample collection varied, with most studies targeting the second trimester (16–27 weeks), although some included samples during labor or in narrower gestational ranges (e.g., 18–22 weeks). Notably, Gu et al. and Jin et al. enrolled large cohorts, while smaller case-control studies, such as Yang et al., focused on highly specific CHD subtypes (Gu et al., 2019; Jin et al., 2021; Yang et al., 2022). Detailed information has been provided in Table 2.

**TABLE 2:** Characteristics of the included studies.

Author, year	Study design	Sample size			Diagnostic method for CHD	CHD type	Gestational week (sample collection)
		Overall	Study	Control			
Gu et al. (2019)	Matched case-control	N = 110	N = 50	N = 60	fetal echocardiography and/or postnatal surgery and/or autopsy	VSD - 13; TOF - 12; SV - 6 PTA-5; OTHER/MIX - 14	STUDY: 26.3 ± 3.4 CONTROL: 26.6 ± 3.8
Jin et al. (2021)	Retrospective cohort study	N = 182	N = 91	N = 91	fetal echocardiography	ALL VSD	STUDY: 16.74 ± 0.50 CONTROL: 16.84 ± 0.46
Kehler et al. (2015)	Cohort study	N = 39	N = 22	N = 17	prenatal ultrasonography	*Not reported*	OVERALL: 22.22 ± 5.20
Kong et al. (2021)	Retrospective cohort study	N = 60	N = 30	N = 30	fetal echocardiography	TOF - 6; VSD - 6; PS - 5; DTGA - 3; DORV - 2; APVC - 2; FSV - 2; RAA - 2; ECD - 1; RVH - 1	DURING OF LABOUR
Xi et al. (2024)	Case-control	N = 72	N = 31	N = 41	fetal echocardiography	VSD - 9; AAA - 6; TOF - 3 TGA - 3; RHLTLH - 3; OTHER- 7	*Not reported*
Yang et al. (2022)	Mixed: animal and case-control	N = 14	N = 7	N = 7	prenatal ultrasonography	ALL VSD	STUDY = 24.59 ± 1.35 CONTROL = 21.80 ± 3.12
Zhu et al. (2013)	Multistage nested case-control study	N = 60	N = 30	N = 30	fetal echocardiography	VSD - 12; TOF - 11; ASD- 4	OVERALL RANGE: 18–22

NOTE: CHD, congenital heart disease; VSD, Ventricular Septal Defect; TOF, Tetralogy of Fallot; SV, Single Ventricle; PTA, Persistent Truncus Arteriosus; PS, Pulmonary Stenosis; DTGA, Dextro-Transposition of the Great Arteries; DORV, Double Outlet Right Ventricle; APVC, Anomalous Pulmonary Venous Connection; FSV, Functional Single Ventricle; RAA, Right Aortic Arch; ECD, Endocardial Cushion Defect; RVH, Right Ventricular Hypertrophy; AAA - Aortic Arch Anomaly; TGA, Transposition of the Great Arteries; RHLTLH, Right Heart Larger Than Left Heart; ASD, Atrial Septal Defect.

The experiments conducted in the included studies utilized diverse biological materials and methodologies to investigate miRNA expression patterns associated with CHD. The majority of studies analyzed maternal serum samples (Zhu et al., 2013; Kehler et al., 2015; Gu et al., 2019; Jin et al., 2021; Xi et al., 2024), while Kong et al. focused on umbilical cord blood and Yang et al. examined amniotic fluid (Kong et al., 2021; Yang et al., 2022). Discovery-phase experiments predominantly employed next-generation sequencing (NGS) technologies, including platforms such as Illumina HiSeq 2500 (Jin et al., 2021), Illumina NextSeq 500 (Xi et al., 2024), and Illumina NovaSeq 6000 (Yang et al., 2022). Zhu et al. utilized SOLiD sequencing, (Zhu et al., 2013), while Gu et al. performed microarray analysis using the miRCURY LNA™ microRNA Array system (Gu et al., 2019).

Targeted validation of miRNA expression was commonly carried out using quantitative reverse transcription PCR (qRT-PCR). SYBR Green systems were employed in most studies, including Gu et al. (2019), Jin et al. (2021), Yang et al. (2022), and Xi et al. (2024) whereas Zhu et al. utilized a TaqMan MicroRNA Assay (Zhu et al., 2013). Notably, Kehler et al. employed a miRCURY LNA™ Universal RT microRNA PCR Kit with LNA-based dye chemistry (Kehler et al., 2015), while Kong et al. used SYBR Green technology with U6 snRNA as an endogenous control (Kong et al., 2021). External controls, such as cel-miR-39, were used in studies by Jin et al., Xi et al., and Zhu et al. to normalize experimental variability (Zhu et al., 2013; Jin et al., 2021; Xi et al., 2024). The detailed experimental workflows and technical systems employed are summarized in Table 3.

**TABLE 3:** Experimental details of included studies.

Author, year	Type of sample	Typ of experiment	Discovery technology used	Targeted technology used	Dye system	Host gene
Gu et al. (2019)	Maternal serum	D+T	Microarray (miRCURY LNA™ microRNA Array)	qRT-PCR (SYBR Premix Ex Taq, ABI 7500 Real-Time PCR System)	SYBR Green	*not reported*
Jin et al. (2021)	Maternal serum	D+T	NGS (Illumina HiSeq 2500)	qRT-PCR (Bulge-loopTM kit, BioRad CFX Real-time PCR System)	SYBR Green	cel-miR-39*
Kehler et al. (2015)	Maternal serum	T	N/A	RT-qPCR (miRCURY LNA™ Universal RT microRNA PCR Kit, Roche LightCycler II)	LNA	U6
Kong et al. (2021)	Umbilical cord blood	T	N/A	qRT-PCR (SYBR Green RT-PCR Kit, CFX96 Real-Time PCR Cycler, TaKaRa One Step PrimeScript miRNA cDNA Synthesis Kit)	SYBR Green	U6
Xi et al. (2024)	Maternal serum	D+T	NGS (Illumina NextSeq 500)	qRT-PCR (Bulge-loopTM kit, Roche LightCycler 480)	SYBR Green	cel-miR-39-3p*
Yang et al. (2022)	Amniotic fluid	D+T	NGS (Illumina NovaSeq 6000)	qRT-PCR (Sangon Biotech MiRNA First Strand cDNA Synthesis, TB Green Premix Ex Taq, Roche LightCycler 480)	SYBR Green	U6
Zhu et al. (2013)	Maternal serum	D+T	NGS (SOLiD)	qRT-PCR (TaqMan MicroRNA Assay, ABI 7500 Real-Time PCR System)	TaqMan	cel-miR-39*

NOTE: * (external control); D - discovery; T–targeted; U6 – Universal U6 snRNA.

### 3.3 Diagnostic potential of identified microRNAs

**FIGURE 2:**
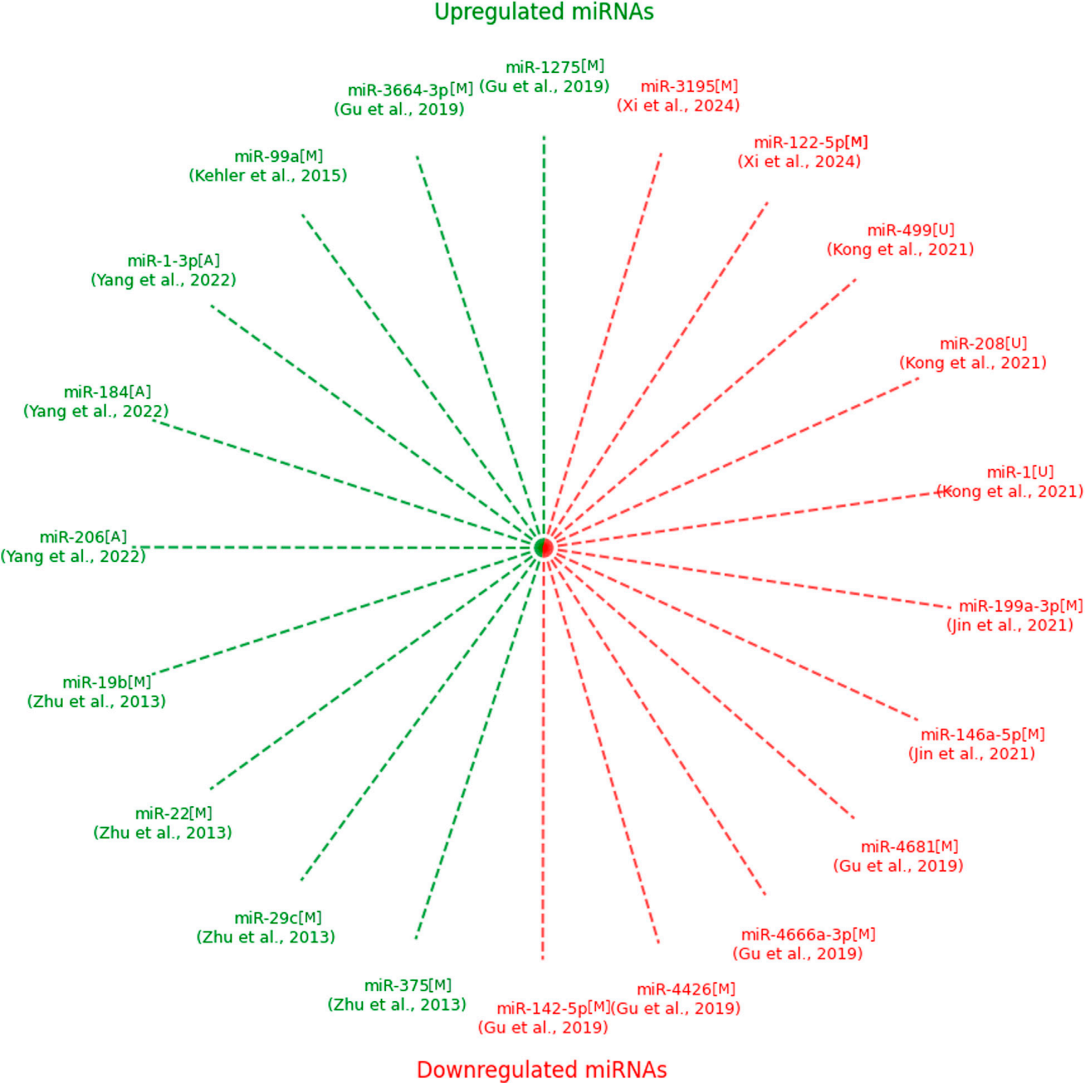
Upregulated and downregulated expressions of microRNAs identified in the included studies with type of sample. NOTE: green–upregulated; red–downregulated; M–maternal serum; U–umbilical cord blood; A–amniotic fluid.

Figure 2 illustrates upregulated (green) and downregulated (red) miRNAs identified in studies evaluating their potential as biomarkers for CHD. Each spoke represents a unique miRNA, with the corresponding study authors indicated in parentheses. Notably, no miRNAs overlapped between studies, which may be attributed to the variability in experimental methodologies, as discussed in the text. Upregulated miRNAs were predominantly observed in studies utilizing maternal serum and amniotic fluid, while downregulated miRNAs were also detected across diverse sample types, including umbilical cord blood. This visualization highlights the heterogeneity in miRNA biomarker discovery for CHD (Figure 2).

Several studies focused on the diagnostic potential of miRNAs by evaluating sensitivity, specificity, and area under the curve (AUC) metrics. Gu et al. reported that miR-142-5p (downregulated) achieved an AUC of 80.4% (95% CI: 72.1%–88.7%), with a combined panel of miR-142-5p, miR-1275, miR-4666a-3p, and miR-3664-3p increasing the AUC to 90.1% (95% CI: 84.0%–96.2%). Other miRNAs analyzed in this study, including miR-1275, miR-4426, and miR-4681, exhibited moderate diagnostic performance with individual AUCs ranging from 66.2% to 71.5% (Gu et al., 2019). Jin et al. demonstrated exceptional diagnostic accuracy for miR-146a-5p (downregulated), achieving sensitivity and specificity of 98.1%, with an AUC of 99.7% (95% CI: 99.2%–100%). Conversely, miR-199a-3p, also downregulated, showed lower performance with an AUC of 67.17% (95% CI: 56.0%–78.3%) (Jin et al., 2021; Table 4).

**TABLE 4:** Diagnostic potential of identified microRNAs.

Author, year	miR	Regulation	Sensitivity (%)	Specificity (%)	AUC (%)	95% CI AUC (%)	COMBO AUC (%)	95CI COMBO AUC (%)
Gu et al. (2019)	miR-142- 5p	down	-	-	80.4	72.1–88.7	90.1*	84.0–96.2*
	miR-1275	up	-	-	71.5	61.5–80.5		
	miR-3664-3p	up	-	-	69.4	59.1–79.7		
	miR-4426	down	-	-	66.2	55.3–77.0		
	miR-4666a-3p	down	-	-	70.3	60.2–80.5		
	miR-4681	down	-	-	66.3	55.5–77.0		
Jin et al. (2021)	miR-146a-5p	down	98.1	98.1	99.7	99.2–100	-	-
	miR-199a-3p	down	58.2	99.9	67.17	56.0–78.3		
Kehler et al. (2015)	miR-99a	up	-	-	-	-	-	-
Kong et al. (2021)	miR-1	down	70	100	86	76–96	-	-
	miR-208	down	53	100	75	63–87		
	miR-499	down	70	100	84	74–95		
Xi et al. (2024)	miR-122-5p	down	-	-	70	57–83	73	60–86
	miR-3195	down	-	-	68	54–81		
Yang et al. (2022)	miR-1-3p	up	-	-	-	-	-	-
	miR-184	up	-	-	-	-	-	-
	miR-206	up	-	-	-	-	-	-
Zhu et al. (2013)	miR-19b	up	74.1	77.8	79.95	66.6–91.4	81.3	69.5–93.1
	miR-22	up	70.4	66.7	67.1	52.5–81.6		
	miR-29c	up	63	88.9	76.7	64.0–89.4		
	miR-375	up	55.6	85.2	69.3	55.0–83.5		

* ONLY: miR-142-5p, miR-1275, miR-4666a-3p and miR-3664-3p.

NOTE: CI, Confidential Interval; AUC, Area Under Curve; COMBO, set of miRNAs.

Kong et al. highlighted the diagnostic capabilities of miR-1, miR-208, and miR-499 (all downregulated). Among these, miR-1 and miR-499 demonstrated the highest AUC values of 86% (95% CI: 76%–96%) and 84% (95% CI: 74%–95%), respectively, with perfect specificity (100%) for all three miRNAs (Kong et al., 2021). Xi et al. identified miR-122-5p and miR-3195 (both downregulated) as potential biomarkers, reporting AUC values of 70% (95% CI: 57%–83%) and 68% (95% CI: 54%–81%), respectively. The combination of these miRNAs improved the diagnostic performance, achieving a combined AUC of 73% (95% CI: 60%–86%) (Xi et al., 2024). In the study by Zhu et al., miR-19b (upregulated) showed the highest sensitivity (74.1%) and specificity (77.8%), with an AUC of 79.95% (95% CI: 66.6%–91.4%). The combined panel of miR-19b, miR-22, miR-29c, and miR-375 increased the AUC to 81.3% (95% CI: 69.5%–93.1%) (Zhu et al., 2013; Table 4).

Other studies primarily reported differences in miRNA expression levels without evaluating their diagnostic performance. For instance, Yang et al. highlighted upregulation of miR-1-3p, miR-184, and miR-206 (Yang et al., 2022), while Kehler et al. described increased expression of miR-99a (Kehler et al., 2015). These findings suggest the potential relevance of these miRNAs in CHD pathology but lack diagnostic validation (Table 4).

### 3.4 Assessment of risk of bias

The risk of bias across the included studies was evaluated using the Newcastle-Ottawa Scale (NOS), which assesses methodological quality across three domains: selection (D1), comparability (D2), and exposure or outcome (D3). Table 5 provides a summary of the results. Among the seven studies assessed, four achieved a “good” rating across all domains, indicating a low risk of bias (Zhu et al., 2013; Gu et al., 2019; Jin et al., 2021; Xi et al., 2024). These studies demonstrated robust methodologies, including adequate selection of study participants, appropriate comparability between groups, and reliable measures of exposure or outcome. Two studies received an “overall middle” rating due to moderate concerns in the domain of comparability (D2) (Kong et al., 2021; Yang et al., 2022). These studies adequately selected participants and measured outcomes but demonstrated limitations in controlling for confounding variables, which may impact the reliability of their findings. Kehler et al. exhibited the highest risk of bias, with ratings of “middle” in selection (D1) and comparability (D2), and “poor” in exposure or outcome (D3) (Kehler et al., 2015). The “poor” rating in D3 suggests potential issues with the accuracy or consistency of exposure or outcome assessment methods, limiting the study’s internal validity. In summary, while most studies were of good methodological quality, potential risks of bias were identified in a minority of studies, particularly in the domains of comparability and exposure or outcome assessment.

**TABLE 5:** Risk of bias assesment using Newcastle-Ottawa Scale.

Author, year	D1	D2	D3	Overall
Gu et al. (2019)	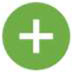	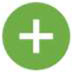	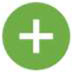	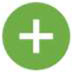
Jin et al. (2021)	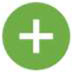	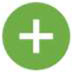	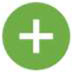	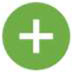
Kehler et al. (2015)	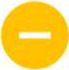	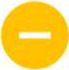	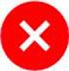	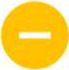
Kong et al. (2021)	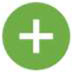	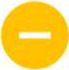	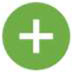	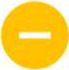
Xi et al. (2024)	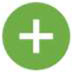	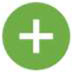	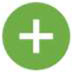	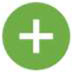
Yang et al. (2022)	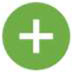	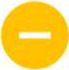	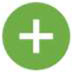	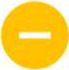
Zhu et al. (2013)	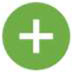	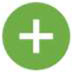	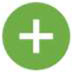	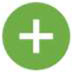

Legend: LOW 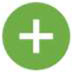 MIDDLE 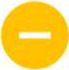 POOR 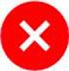

D1 - Domain: selection; D2 - Domain: comparability; D3 - Domain: exposure/outcome.

## 4 Discussion

The seven studies included in this analysis collectively present promising evidence that specific miRNAs may serve as non-invasive biomarkers for the early detection of CHD in fetuses. However, the findings also reveal substantial heterogeneity in study designs, methodologies, and experiments, which necessitates cautious interpretation and limits the generalizability of the results.

These seven studies involved different miRNAs for CHD, and the studies followed different methodologies to assess the potential of miRNAs as biomarkers. Zhu et al. identified miR-19b, miR-22, miR-29c, and miR-375 as potential biomarkers. The AUC was 81.3%, which is a good diagnostic accuracy (Zhu et al., 2013). Gu et al. also found high AUC values of 0.920 for miR-142-5p, miR-1275, miR-4666a-3p, and miR-3664-3p that effectively discriminated the cases from controls (Gu et al., 2019). Kong et al. identified potential biomarkers of miRNA-1, miRNA-208, and miRNA-499 in umbilical cord blood, with miRNA-1 showing the highest AUC of 0.86 (Kong et al., 2021). Jin et al. mentioned that miR-146a-5p could predict VSDs moderately. Kehler et al. and Yang et al. went ahead to identify certain miRNAs related to CHD and, hence, established the possibility of miRNAs as biomarkers (Kehler et al., 2015; Yang et al., 2022).

Overall, it is indicated that miRNAs bear great potential as non-invasive biomarkers for prenatal CHD diagnosis. The high AUC reported in many studies suggests that some miRNAs may be able to distinguish between fetuses with and without CHD effectively. This is significant for early detection which may lead to improved clinical outcomes by timely intervention. However, considering the variability in the miRNAs studied and the differences in the populations, sample sizes, and methodologies among the studies, these results should be interpreted cautiously. The heterogeneity in these studies can partly be attributed to the variable timing of sample collection, the type of CHD addressed, and the methodology for miRNA detection and quantification. These may account for the variability in the diagnostic performance seen across the different studies.

The clinical significance of circulating miRNAs as markers for detecting CHD remains an area of ongoing research. While miRNAs hold promise, several challenges and limitations need to be addressed in their application. CHD encompasses a wide range of structural heart abnormalities, each with distinct genetic and molecular underpinnings. The diversity of CHD phenotypes complicates the identification of specific miRNAs that universally correlate with all types of CHD (de Gonzalo-Calvo et al., 2022).

MiRNA expression can vary significantly among individuals and tissues. Identifying consistent miRNA signatures across diverse CHD cases is challenging due to this variability. There is a lack of standardized methods for miRNA detection and quantification. Consistent protocols are needed to ensure reliable and reproducible results across different laboratories. Environmental influences and genetic factors can impact miRNA expression levels. Understanding these confounding factors is essential for accurate interpretation of miRNA data. While circulating miRNAs offer potential as diagnostic markers for CHD, addressing these challenges will enhance their clinical utility.

### 4.1 Strengths and limitations

The strengths of this review are underscored by its stringent methodology, comprehensive literature search, and systematic assessment of bias using the Newcastle-Ottawa Scale. By exclusively including studies with well-defined inclusion criteria, the reliability of the results was further bolstered, allowing for the evaluation of multiple miRNAs across various types of CHD.

However, several limitations must be acknowledged. Many of the included studies featured small sample sizes, which may compromise the generalizability of the findings. In addition, the studies employed diverse experimental methodologies for isolating and measuring miRNAs, contributing to variability in the reported results. The heterogeneity of biological materials analyzed, including maternal serum, amniotic fluid, and umbilical cord blood, further complicates the interpretation and synthesis of data. These differences preclude the possibility of conducting a comprehensive synthesis of results or a meta-analysis.

Moreover, CHDs represent a heterogeneous group of disorders with varying etiologies and pathophysiological mechanisms. Consequently, drawing generalized conclusions regarding the entire spectrum of CHDs based on the available data may introduce bias and lead to erroneous assumptions. These factors necessitate a cautious interpretation of the results and underscore the need for future studies with larger sample sizes, standardized methodologies, and a focus on specific CHD subtypes to enhance the reliability of findings related to miRNA biomarker utility in prenatal CHD diagnosis.

## 5 Conclusion

The findings of this systematic literature review provide evidence that some miRNAs could serve as non-invasive biomarkers for the early detection of CHD in fetuses. However, each of the reviewed studies identified different miRNAs as potential biomarkers. This variability may stem from differences in experimental methodologies, including approaches to miRNA isolation, quantification techniques, and the types of biological materials analyzed. Such methodological heterogeneity, combined with small sample sizes and the diverse spectrum of CHDs, underscores the need for caution in interpreting these findings.

At this stage, it is not feasible to translate these results into clinical practice or establish standardized miRNA-based prenatal screening protocols. Further research involving larger, well-designed studies with standardized methodologies and a focus on specific CHD subtypes is essential to validate these preliminary findings and explore their clinical applicability.
